# Diversity and antimicrobial activity of the tropical ant-derived actinomycetes isolated from Thailand

**DOI:** 10.3934/microbiol.2024005

**Published:** 2024-01-22

**Authors:** Tuangrat Tunvongvinis, Weeyawat Jaitrong, Yudthana Samung, Somboon Tanasupawat, Wongsakorn Phongsopitanun

**Affiliations:** 1 Department of Biochemistry and Microbiology, Faculty of Pharmaceutical Sciences. Chulalongkorn University, Bangkok 10330, Thailand; 2 Office of Natural Science Research, National Science Museum, 39, Moo 3, Khlong 5, Khlong Luang, Pathum Thani 12120, Thailand; 3 Department of Medical Entomology, Faculty of Tropical Medicine, Mahidol University, Bangkok 10400, Thailand; 4 Natural Products and Nanoparticles Research Units (NP2), Chulalongkorn University, Bangkok 10330, Thailand

**Keywords:** Actinomycetes, antimicrobial activity, social insect, ants

## Abstract

Antibiotic resistance is one of the most important global healthcare challenges and is responsible for the mortality of millions of people worldwide every year. It is a crisis attributed to misuse of antibiotics and a lack of new drug development. Actinomycetes constitute a group of Gram-positive bacteria known for their distinctive high guanine-cytosine (G+C) content in their genomic DNA. These microorganisms are widely recognized for their capability to generate a wide range of secondary metabolites with diverse biological activities. These versatile microorganisms are ubiquitous in diverse ecosystems, including soil, freshwater, marine sediments, and within the bodies of insects. A recent study has demonstrated that social insects, such as ants, host a diverse array of these bacteria. In this study, we involved the isolation and characterization of a total of 72 actinomycete strains obtained from 18 distinct ant species collected from various regions across Thailand. Utilizing 16S rRNA gene analysis, these isolated actinomycetes were classified into four distinct genera: *Amycolatopsis* (2 isolates), *Micromonospora* (1 isolate), *Nocardia* (8 isolates), and *Streptomyces* (61 isolates). Among the *Streptomyces* strains, 23 isolates exhibited antimicrobial activity against a panel of Gram-positive bacteria, including *Bacillus subtilis* ATCC 6633, *Staphylococcus epidermidis* ATCC 12228, *Staphylococcus aureus* ATCC 25923, *Kocuria rhizophila* ATCC 9341, and Methicillin-resistant *Staphylococcus aureus* (MRSA) DMST 20646. Additionally, two isolates displayed antifungal activity against *Candida albicans* TISTR 5554. Based on 16S rRNA gene sequence similarity studies, these two isolates, ODS25 and ODS28, were demonstrated to be closely related to *Streptomyces lusitanus* NBRC 13464^T^ (98.07%) and *Streptomyces haliclonae* DSM 41970^T^ (97.28%), respectively. The level of 16S rRNA gene sequence similarity below 98.65% cutoff indicates its potential as a novel actinomycete species. These findings underscore the potential of actinomycetes sourced from ants as a valuable reservoir of novel antimicrobials.

## 1. Introduction

Actinomycetes are Gram-positive bacteria with high guanine-cytosine (G+C) content. Actinomycetes are the source of many compounds commonly used clinically in antibiotics, such as streptomycin, erythromycin [1], tetracycline, vancomycin, and neomycin, for treating a wide range of bacterial infections. Additionally, actinomycete-derived compounds such as actinomycin D and bleomycin are used in treating various cancers. Further, an actinomycete-derived compound, clavulanic acid [2], can be combined with other antibiotics to treat microbial infections. Rapamycin [3], an additional significant compound derived from actinomycetes, holds notable importance as an immunosuppressant employed in the prevention of organ transplant rejection and the treatment of specific cancer types. [1],[4],[5].

Antimicrobial resistance (AMR) is an increasingly concerning issue worldwide. This happens when microorganisms such as viruses, parasites, bacteria, and fungi become resistant to antimicrobial agents that were previously effective in treating infections caused by such organisms [6],[7]. The World Health Organization (WHO) has listed antimicrobial resistance (AMR) as one of the top ten dangers to global public health. Recent research has extensively documented the emergence of antimicrobial resistance in pathogenic microorganisms, posing a significant challenge to global healthcare in the 21st century. The resistance of bacteria to commonly used antibiotics has led to severe infections, posing a substantial threat to public health. Examples of some of the most resistant bacteria causing significant community-acquired infections such as methicillin-resistant *Staphylococcus aureus* (MRSA), vancomycin-resistant *Staphylococcus aureus* (VRSA), vancomycin-resistant *Enterococcus* (VRE), and multiple drug-resistant *Mycobacterium tuberculosis* (MDR-MTB) [8].

Therefore, researchers are actively searching for new, safe, and effective antimicrobial agents to fight against drug-resistant infections, and natural sources are a potential avenue for exploration. Actinomycetes have gained attention as a promising source for new antibiotics due to their vast potential for producing biologically active compounds [9]. To improve the chances of discovering new molecules with unique mechanisms of action, the focus should shift away from commonly explored sources like soil bacteria and towards less investigated natural sources [10],[11]. Insects are one potential source of new antimicrobial compounds. Research papers investigating insect-derived antimicrobial compounds have risen steadily in the past decade [12],[13]. The investigation of insect antimicrobial peptides and those synthesized by their symbiotic microorganisms constitutes a prominent domain within drug discovery research. [13],[14]. Recently, Chevrette and their colleagues conducted a study using genomic analysis, metabolomics, and bioactivity assays to examine *Streptomyces* strains associated with insects to investigate their potential as a source of novel antimicrobial drugs. According to Chevrette et al., *Streptomyces* derived from insects exhibited a substantial number of unidentified biosynthetic gene clusters, which led to the discovery of new compounds with significantly higher antifungal efficacy than *Streptomyces* derived from soil [15]. This finding suggests that investigating the chemical properties of insect-associated actinomycetes could be a promising strategy for discovering novel chemotypes with antimicrobial activity.

Ants are eusocial insects that form complex colonies with specialized roles for each member. These colonies are often dominated by a queen, who reproduces and is attended by sterile workers. They can be found in a variety of land-based environments and their nests can range from simple holes in the ground to elaborate systems of tunnels and chambers. These nests serve as their homes and provide shelter, protection, and a place to raise their young. Ants can foster the growth of fungi, which poses a significant risk to both their offspring and their food. Nevertheless, ants have managed to survive in such conditions by devising various defensive tactics [16],[17]. Thus, one of the most well-studied examples of ant symbiosis is the relationship between ants and actinomycetes [18]. Some ants are thought to have evolved a symbiotic relationship with actinomycetes to protect themselves from pathogens and parasites. These actinomycetes play a critical role in ant health and colony dynamics. Moreover, actinomycetes associated with ants has the capability to produce a broad spectrum of bioactive compounds that help ants defend against pathogens and parasites, regulate their immune systems, and aid in nutrient cycling and other ecological processes [19].

Therefore, in this study, we aimed to identify new sources of antimicrobials by examining actinomycetes from insect microbiomes in 18 species of ants, including *Harpegnathos venator* (Smith, 1858); *Acanthomyrmex ferox* Emery, 1893; *Aphaenogaster feae* Emery, 1889; *Anochetus graeffei* Mayr, 1870; *Camponotus lasiselene* Wang & Wu, 1994; *Centromyrmex feae* (Emery, 1889); *Ectomomyrmex astutus* (Smith, 1858), *Echinopla striata* Smith, 1857; *Gnamptogenys coxalis* (Roger, 1860); *Liopnera* sp., *Polyrchachis laevissima* Smith, 1858; *Odontomachus simillimus* Smith, 1858; *Parasyscia* sp., *Diacamma orbiculatum* Santschi, 1932; *Stigmatomma reclinatum* (Mayr, 1879); *Syscia chaladthanyakiji* Jaitrong, Wiwatwitaya & Yamane, 2020; *Tetraponera nigra* (Jerdon, 1851); and *Tetraponera rufonigra* (Jerdon, 1851), using culture-dependent and antimicrobial activity screening methods.

## 2. Materials and methods

### 2.1. Sample collection and isolation method

**Figure 1.**
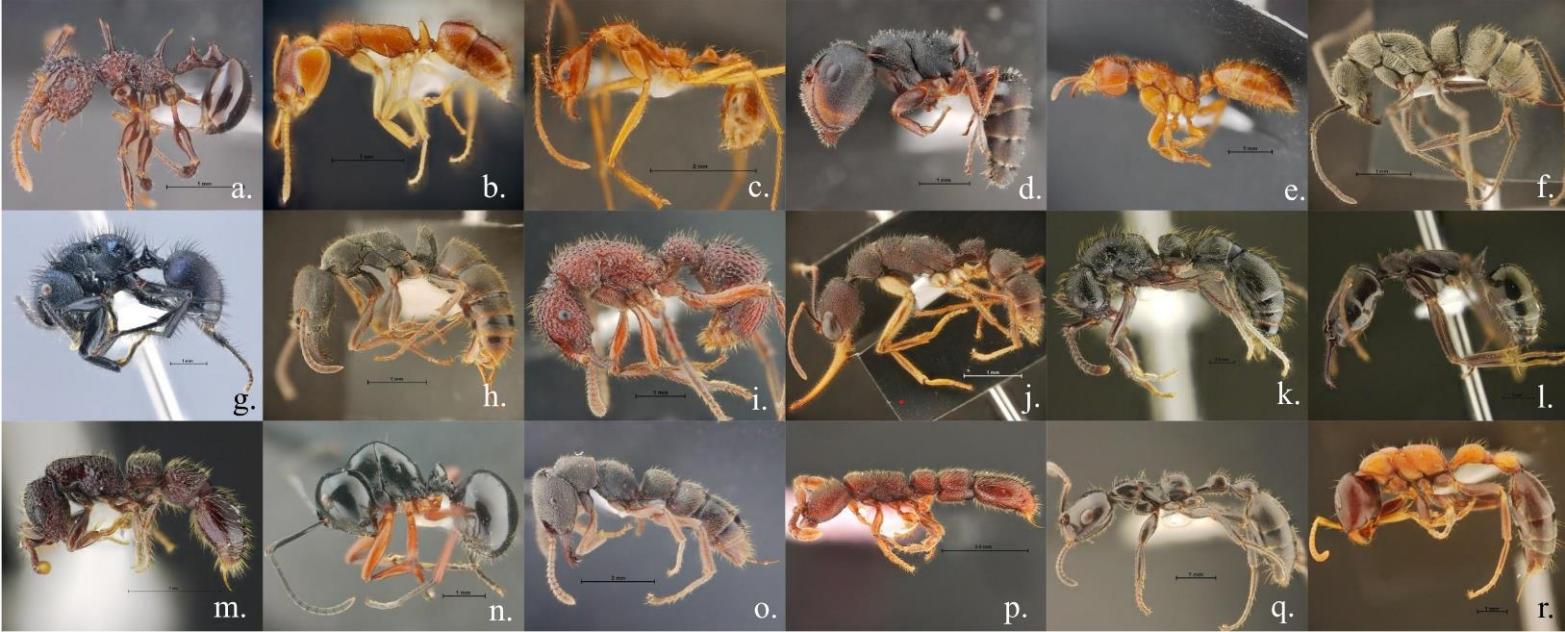
The pictures illustrated the ant sample used in this study including a. *Acanthomyrmex ferox* b. *Anochetus graeffei* c. *Aphaenogaster feae* d. *Camponotus lasiselene* e. *Centromyrmex feae* f. *Diacamma orbiculatum* g. *Echinopla striata* h. *Ectomomyrmex astutus* i. *Gnamptogenys coxalis* j. *Harpegnathos venator* k. *Liopnera* sp. l. *Odontomachus simillimus* m. *Parasyscia* sp. n. *Polyrchachis laevissima* o. *Stigmatomma reclinatum* p. *Syscia chaladthanyakiji* q. *Tetraponera nigra* r. *Tetraponera rufonigra*.

**Figure 2.**
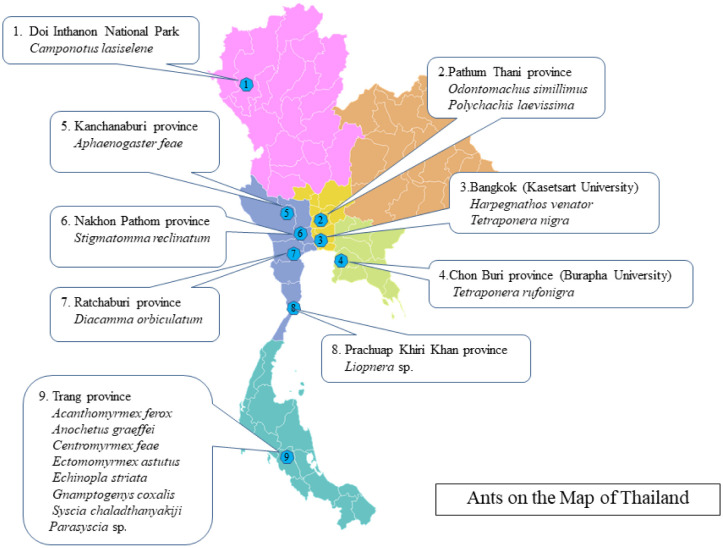
The location for ant samples collection.

Ant samples were gathered from various locations in Thailand, which included Kasetsart University (Bangkok), Burapha University (Chon Buri), Doi Inthanon National Park, Pathum Thani, Kanchanaburi, Nakhon Pathom, Ratchaburi, Prachuap Khiri Khan, and Trang provinces (Figures 1 and 2). Sterile forceps were used to collect the ant samples, which were placed in sterilized containers and stored in a refrigerator at −20 °C until processing. The isolation of actinomycetes in each ant involved two steps. First step, we examined the ant exoskeletons for the presence of actinomycetes. The ant samples underwent a thorough washing process to eliminate adherent epiphytes and surface soil by placing them into an Eppendorf tube filled with 1.2 mL of sterile water, followed by vortexing for 30 seconds and gently rubbing each sample against the surface of the growth media [20]. In this study, four different media, including humic acid vitamin agar [21], starch casein nitrate agar [22], proline agar [23], and soil extract agar [24], were used for the actinomycetes isolation. All media were supplemented with nalidixic acid (25 mg/L) and cycloheximide (50 mg/L) to suppress the growth of Gram-negative bacteria and fungi.

In the second step, we sought actinomycetes within each sample. To isolate actinomycetes within the ant bodies, two to five worker ant samples were thoroughly washed with sterile water and then crushed using a sterile mortar and pestle. The crushed samples were mixed with 500 µL of a basic lauryl-sulfate solution, followed by ten-fold serial dilution. Then, each dilution (0.1 mL) was spread on four different types of media [20]. Additionally, the ants' wash, obtained during the final washing process, underwent testing for the successful removal of the exoskeleton microbe. Point one milliliter of the ants' wash was spread onto each of the four different media. The plates were incubated at 30 °C for 14 days. Colonies grown on the agar plates were observed under a light microscope and then transferred to an ISP 2 medium [25] for purification.

### 2.2. Identification of actinomycetes

Identification of actinomycetes was conducted by analyzing the 16S rRNA gene. Genomic DNA for the 16S rRNA was extracted from cells grown in yeast extract-malt extract broth under shaking conditions at 180 rpm at 30 °C for 3–7 days [26]. Genomic DNA was extracted using DNA extraction kits (Purelink™). The 16S rRNA gene was amplified using two primers: the 20F primer (5′-GAGTTTGATCCTGGCTCAG-3′) and 1500R (5′-GTTACCTTGTTACGACTT-3′). The polymerase chain reaction (PCR) mixture consisted of 4 µL of each primer (10 pmol/µL), 2 µL of dNTP (10 mM), 10 µL of 10x *Taq* buffer, 8 µL of MgCl (25 mM), 0.5 µL of *Taq* DNA polymerase, 61.5 µL of dH_2_O, and 10 µL of template DNA, resulting in a final volume of 100 µL. The PCR conditions involved an initial denaturation at 94 °C for 3 minutes, followed by 30 cycles with denaturation at 94 °C for 1 minute, annealing at 50 °C for 1 minute, and extension at 72 °C for 3 minutes [27]. A PCR purification kit (Gene aid) was used for purifying the PCR product, and nucleotide sequencing was carried out using universal primers 27F, 518F, 800R, and 1492R [28]. Sequencing services were provided by Macrogen (Korea). The nucleotide sequences were analyzed manually using BioEdit software (Ibis Biosciences), and BLAST analysis was performed using the EzbioCloud database [29]. For phylogenetic analysis, MEGA X software [30] was employed, and the tree topology was evaluated using the bootstrap test [31]. A set of 72 isolated actinobacterial 16S rRNA gene sequences, 41 closely related type strains and one outgroup were used for phylogenetic analysis. The accession numbers of the sequence used in this study are provided in Figure 3a and Table S1. *Bacillus subtilis* JCM 1465^T^ was used as the outgroup in the tree.

### 2.3. Screening of antimicrobial activities

The disc diffusion method [32] was employed to screen for antimicrobial activity. Actinomycetes were cultivated in ISP 2 broth with shaking at 180 rpm at 30 °C for 14 days. At the end of fermentation, each 10 mL of culture liquid, including cells, was extracted for the secondary metabolites by adding an equal volume of 95% ethanol and shaking at 180 rpm at 30 °C for 2 hours. Subsequently, the cell suspension was centrifuged at 775 g for 15 minutes, and the supernatant was collected and stored at −20 °C for testing. The medium used for preparing for the bacterial tests was Mueller Hinton agar (MHA). Paper discs (6 mm) were drenched with 50 µL of the supernatant and dried at room temperature. These discs were then placed on the surface of agar plates containing pathogenic bacteria, including *B. subtilis* ATCC 6633, *S. epidermidis* ATCC 12228, *S. aureus* ATCC 25923, *K*. *rhizophila* ATCC 9341, Methicillin-resistant *S. aureus* (MRSA) DMST 20646, *Streptococcus pyogenes* DMST 4369, *Klebsiella pneumoniae* ATCC 13883, *Klebsiella aerogenes*, *Escherichia coli* ATCC 25922, *Pseudomonas aeruginosa* ATCC 27853, *Salmonella typhi* (clinical isolate), and *Shigella* sp. (clinical isolate). The plates were then incubated at 37 °C for 24 hours. The positive control used was gentamycin (30 mg) and vancomycin (30 mg) for MRSA and *S. aureus*, while the negative control consisted of ethanol in the media solution. Antifungal activity screening was conducted against *Candida albicans* TISTR 5554, *Candida guilliermondii* TISTR 5206, *Candida glabrata* TISTR 5006, *Candida tropicalis* TISTR 5268, *Candida parapsilosis* TISTR 5007, and *Candida pseudotropicalis* TISTR 5336 using the disc diffusion method, similar to the microbial screening, but with a deferred medium on sabouraud dextrose agar (SDA). Incubation was carried out at 30 °C for 14 days. The positive control was nystatin (50 mcg), and the negative control was ethanol in the media solution. The inhibition zone (mm) was measured using a Vernier caliper.

## 3. Results

In this study, we aimed to explore actinomycete sources in ants, with a specific focus on both the exoskeletons and internal bacteria. No actinomycete isolates were found in the ant exoskeletons and ants' wash; however, the extraction of bacteria from the internal parts of ants was successful. Actinomycetes were isolated from 18 ant species, resulting in a total of 72 actinomycete strains (Table S1). These actinomycete isolates were obtained from four different types of media including humic acid vitamin agar (38 isolates), starch casein nitrate agar (16 isolates), proline agar (13 isolates), and soil extract agar (5 isolates). Among ant species, most actinomycete isolates were obtained from *C. lasiselene* (18 isolates) followed by *O. simillinus* (11 isolates), *A. graeffei* (6 isolates) and *D. orbiculatum* (6 isolates), respectively. Based on BLAST and phylogenetic tree analysis, the resulting actinomycete isolates were classified into four genera: *Amycolatopsis* (2 isolates), *Micromonospora* (1 isolate), *Nocardia* (8 isolates), and *Streptomyces* (61 isolates) (Figure 3a; Table S1). The predominant genus found in ant samples was *Streptomyces* (85%), followed by *Nocardia* (11%), *Amycolatopsis* (3%), and *Micromonospora* (1%) (Figure 3b). All details of the BLAST result and 16S rRNA gene similarity is provided in Table S1. The antimicrobial activity of the 72 actinomycete strains was assessed against various microorganisms, including six Gram-positive bacteria (*B. subtilis, S. aureus, K. rhizophila, S. epidermidis*, methicillin-resistant *S. aureus* (MRSA), and *S. pyogenes*), six Gram-negative bacteria (*K. pneumoniae*, *K. aerogenes*, *E. coli, P. aeruginosa*, *S. typhi*, and *Shigella* sp.), and six yeast species (*C. albicans*, *C. guilliermondii*, *C. glabrata*, *C. tropicalis*, *C. parapsilosis*, and *C. pseudotropicalis*). Among actinomycete isolates, *Nocardia*, *Amycolatopsis*, and *Micromonospora* isolates exhibited no antimicrobial activity against the tested microorganisms.

All actinomycetes obtained in this study do not exhibit antimicrobial activity against the tested Gram-negative bacteria. The lack of inhibitory activity against Gram-negative bacteria is influenced by their innate features, including an outer membrane that acts as a robust barrier [34]. Gram-negative bacteria employ diverse resistance mechanisms, such as deactivating enzymes and alterations in antibiotic targets, which bolster their resilience [35]. The double-membrane structure hinders antibiotic efficacy by impeding specific actions [36]. In contrast, Gram-positive bacteria, with thicker peptidoglycan layers, are more easily inhibited due to their structural composition, which facilitates antibiotic absorption [37]. However, out of the tested strains, only 23 exhibited antimicrobial activity against Gram-positive bacteria and yeasts. These active strains were derived from nine ant species: *Harpegnathos venator*, *Anochetus graeffei*, *Camponotus lasiselene*, *Lioponera* sp., *Polyrchachis laevissima*, *Odontomachus simillimus*, *Parasyscia* sp., *Diacamma orbiculatum*, and *Tetraponera nigra*. The active strains were identified as *Streptomyces aculeolatus* (CL25-3), *Streptomyces ardesiacus* (RAB12), *Streptomyces bikiniensis* (HPV06), *Streptomyces costaricanus* (CL12, CL20, TEN01), *Streptomyces lusitanus* (ODS25), *Streptomyces marinus* (ODS28), *Streptomyces microflavus* (PA03), *Streptomyces murinus* (CL17), *Streptomyces olivaceus* (CL02, CL05, CL10, CL11, CL18, CL19, CL23), *Streptomyces parvulus* (AG05, LKA04, LKA08, ODS20), *Streptomyces sioyaensis* (ODS18), and *Streptomyces tendae* (LI03).

*Streptomyces costaricanus* (CL12) and *S. murinus* (CL17) demonstrated potent antifungal activity against a variety of pathogens, including 10 out of 18 tested (Table S1). Esnard et al. [33] studied the *S. costaricanus* strain CR-43, isolated from tropical soil, and found that it exhibits antinematodal activity against *Caenorhabditis elegans* and antifungal activity against *Rhizoctonia solani* and *Elhiurn aphunidermatum*, which are soilborne necrotrophic pathogens. Additionally, they found that *S. costaricanus* strain CR-43 was similar to the six species and subspecies, *S. hygroscopicus*, *S. hygroscopicus* subsp. *decoyicus*, *S. griseoluteus*, *S. rubiginosus*, and *S. griseofuscus*, which included *S. murinus*. Ge et al. [34] reported that the *S. murinus* JKTJ-3 strain exhibited antifungal properties against *Pythium aphanidermatum* (Pa). Several *Pythium* species can cause seed decay, damping-off, cutting, and plant stem rot. The study unveils insight using *S. murinus* as a biocontrol agent against Pa-induced watermelon damping-off. Furthermore, the investigation demonstrated that *S. murinus* possessed the capability to produce substances, including chitinase and actinomycin D, to counter oomycetes recognized as formidable plant pathogens impact plant health and agricultural ecosystems.

Seven out of nine strains of *S. olivaceus* were active against different pathogens (Table S1). However, all were obtained from the same species of ants because the intricate dynamics of actinomycete communities within ant species indicate a complex interplay of ecological factors, particularly with the recurrence of species within a single ant species. Environmental conditions, host-specific traits, and microbial interactions contribute significantly to the observed diversity. Moreover, the investigation of antimicrobial production among isolated *Streptomyces* highlights the dynamic nature of microbial secondary metabolite expression. The variability in antimicrobial capabilities is proposed to be influenced by a combination of genetic heterogeneity, environmental stimuli, and co-evolutionary processes with the ant host [20].

Four out of five *S. parvulus* (AG05, LKA04, LKA08, ODS20) were active against at least 6 pathogens. These *S. parvulus* were isolated from different species of ants collected in different areas of Thailand. In fact, *S. parvulus* is found in diverse habitats such as marine sediment, terrestrial soil, and symbioses with insects and plants. The study of *S. parvulus* is important due to the recognition of the importance of *S. parvulus* in the production of actinomycin D [35]–[37]. Actinomycin D from *S. parvulus*, a well-established member of the actinomycin group, is recognized for its potent antibacterial and anticancer characteristics [38]. Shetty et al. [39] reported that *S. parvulus* strain RSPSN2 synthesized a polypeptide antibiotic (actinomycin D) against streptomycin-resistant bacteria *such as B. cereus, P. mirabilis*, *and P. putida*. Moreover, actinomycin D is a key treatment for tumors like Wilms tumor and Ewing sarcoma [40]. It also holds significance in managing choriocarcinoma and testicular tumors [40]. According to Somphong et al. [41] demonstrated that *S. parvulus* isolated from *Pyxine cocoas* lichen, produces an antibiotic with potent antimicrobial and antitumor properties. In addition to the importance of actinomycin D as an antibacterial properties, Chandrakar and Gupta documented the antifungal properties of actinomycin D against *Candida albicans* and *Aspergillus niger* [42].

Last, based on the 16S rRNA gene sequence similarity showed that ODS25 was closely related to *Streptomyces lusitanus* NBRC 13464^T^ (98.07%) and ODS28 was closely related to *Streptomyces haliclonae* DSM 41970^T^ (97.28%). A 16S rRNA gene sequence similarity lower than 98.65% is recognized as potential identification as a novel actinomycete species [43]. Thus, these two isolates are considered candidates for novel actinomycete species, but the full taxonomic descriptions need to be investigated in future study.

**Figure 3 (a).**
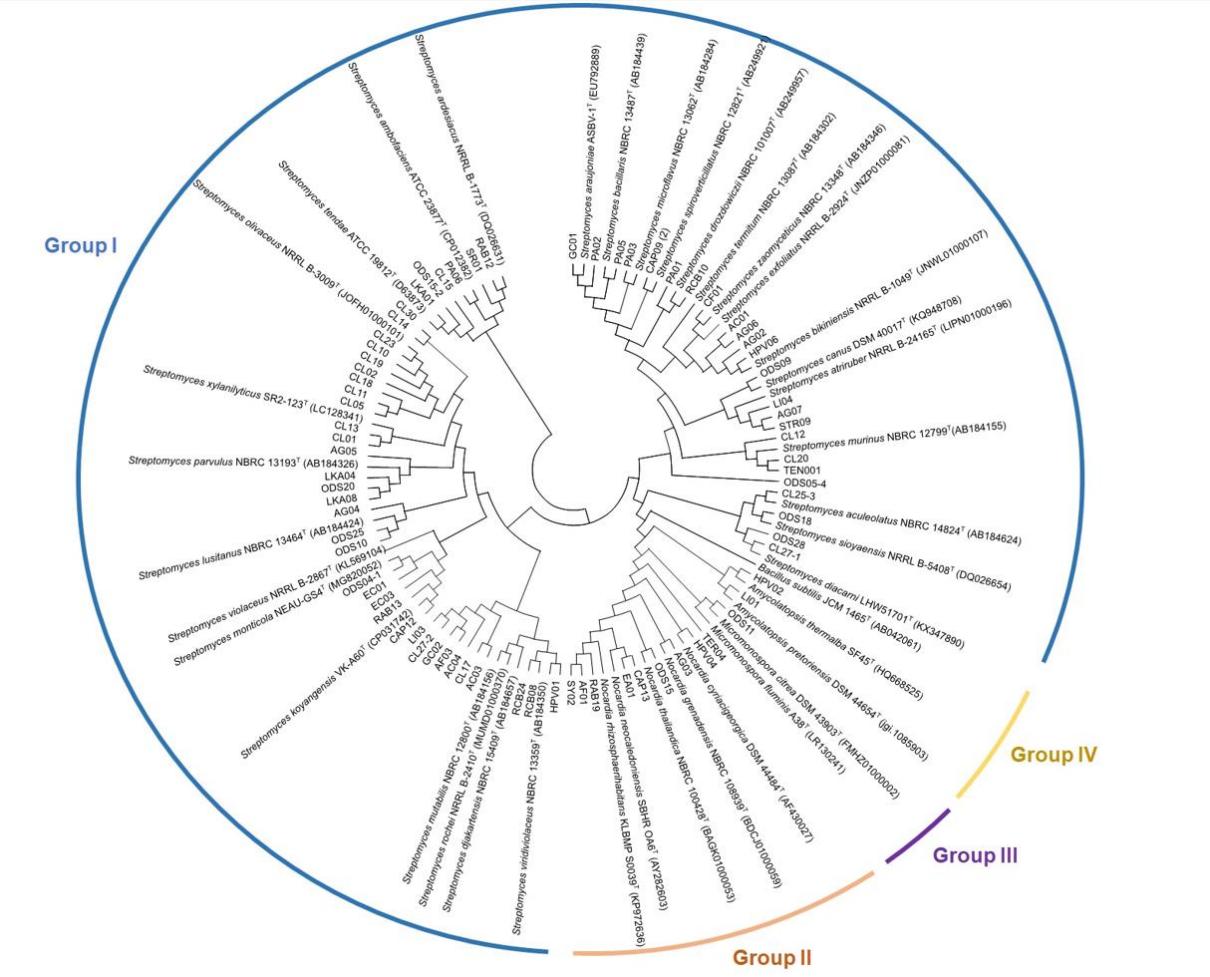
Shows a neighbor-joining phylogenetic tree constructed from 16S rRNA gene sequences of 72 actinomycetes and their closely related type strains, demonstrating the relationship of the isolates separated into groups I (*Streptomyces*), II (*Nocardia*), III (*Micromonospora*), and IV (*Amycolatopsis*).

**Figure 3 (b).**
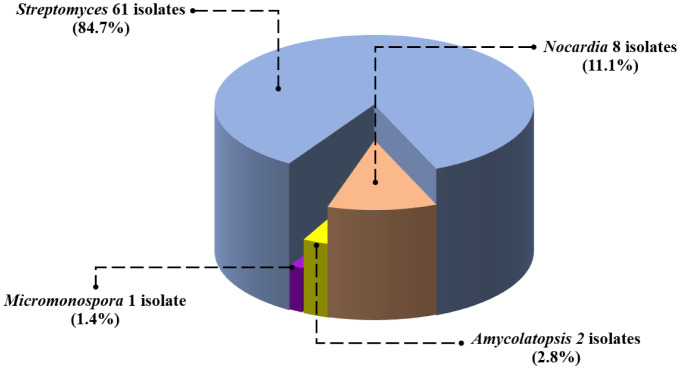
Presents a pie chart illustrating the ratio of actinomycete isolates by genera.

## 4. Discussion

The coevolution between fungus-gardening (attine) ants and microbes has been investigated for more than a decade [44]. However, the study of ant-associated actinomycetes in tropical ant species remains limited. In this study, we explored the differences of culturable ant-associated actinomycetes from 18 ant species collected in various locations across Thailand. Our focus was on isolating actinomycetes from ants and evaluating their antimicrobial potential against a range of microorganisms.

Our findings unveiled a diverse array of actinomycete strains associated with ants, representing genera such as *Streptomyces*, *Amycolatopsis*, *Micromonospora*, and *Nocardia*. The wide variety of actinomycetes found in ants aligns with prior research highlighting the abundant microbial communities present in insect hosts [45]. Particularly noteworthy is that most strains belonged to the *Streptomyces* genus, renowned for its prolific production of bioactive secondary metabolites [1],[46]. The prevalence of *Streptomyces* species in ant samples mirrors previous reports that found this pattern in several ant species, including *Pseudomyrmex penetrator*, *Petalomyrmex phylax*, and *Crematogaster margaritae*, as well as *Acromyrmex subterraneus brunneus* [47],[48].

We successfully isolated actinomycetes from all ant samples. However, it is worth noting that all of these isolates were obtained from the entire ant after the washing process. No isolates were obtained from direct streaking on the exterior of the ant samples. This indicates that these actinomycetes are not located on the outside of the ant's exoskeleton. This distinction contrasts with fungus-growing ants, where actinomycetes can be found outside the ant's body [49]. It is important to emphasize that the limitation of our study is that all ant samples consist of worker ants. However, this finding may be a clue that the diversity of actinomycetes in ants may depend on the ant behavior because all ant species used in this study are not fungus-growing ants.

Notably, most actinomycete isolates were obtained from *Camponotus lasiselene*. This high diversity of actinomycetes in *C. lasiselene* is consistent with previous reports. In the past decade, several new actinomycete species have been isolated from *Camponotus* ants, including *Amycolatopsis camponoti* from *Camponotus vagus* [50]*, Streptomyces capitiformicae*, *Streptomyces camponoticapitis*, *Streptomyces formicae* and *Nocardia camponoti* from the heads of *Camponotus japonicus* [51]–[54], as well as *Promicromonospora alba* isolated from the cuticle of *C. japonicus* [55]. Furthermore, some bioactive compounds were also isolated from the bacteria isolated from *Camponotus* ant such as nybomycin from *Streptomyces* isolated from *C. vagus* [56], deinococcucins A-D, aminoglycolipids, from *Deicococcus* sp. isolated from gut of *C. japonocus* [57] and *Streptomyces* sp. 1H-GS5 isolated from head of *C. japonocus* produces cytotoxic spectinabilin derivative [58].

In general, a 16S rRNA gene sequence similarity of 98.65% is often used as the threshold for differentiating two bacterial species [43]. In this study, two isolates, ODS25 and ODS28, obtained from *O. simillinus*, displayed only 98.07% and 97.28% 16S rRNA gene similarity related to *Streptomyces lusitanus* NBRC 13464^T^ and *Streptomyces haliclonae* DSM 41970^T^, respectively. Therefore, these two isolates are considered candidates for novel actinomycete species. However, this hypothesis will require confirmation through a polyphasic approach in future studies. Based on this finding and the previous literature, it seems that ants are one of the promising sources for finding new actinomycetes.

In our study, when screening the isolated strains against a panel of microorganisms, we discovered promising antimicrobial activity in 23 *Streptomyces* strains against Gram-positive bacteria and yeasts. This finding highlights the potential of ants as a source of bioactive compounds exhibiting antimicrobial properties. Furthermore, ants from various genera, including *Harpegnathos*, *Anochetus*, *Camponotus*, *Lioponera*, *Polyrchachis*, *Odontomachus*, *Parasyscia*, *Diacamma*, and *Tetraponera*, hosted these active strains. The bioactive compounds produced by these *Streptomyces* strains may impact drug discovery and therapeutic development significantly.

The concept of “new microbe-new bioactive compounds” is also found in ant-derived actinomycetes. The good example is *Streptomyces formicae* isolated from *Tetraponera penzigi* could produce 16 new molecules including fasamycins A-E and formicamycins A-M which exhibit antibacterial activity against *B. subtilis*, MRSA and vancomycin resistant enterococci (VRE) [59]. *Streptomyces* sp. BA01 isolated from a gut of wood ant (*Formica yessensis*) produces two new macrolides, formicolides A and B. Both compounds induced quinone reductase activity in murine Hepa-1c1c7 cells and exhibited antiangiogenic activity [60]. *Streptomyces* sp. STA1 isolated from the gut of carpenter ant (*Camponotus kiusiuensis*) produce new polyketide alkaloids named camporidines A and B with antimetastatic and anti-inflammatory activity [61]. We believe our two novel strains (ODS25 and ODS28) might produce new compounds, but the intensive study of their bioactive metabolites has to be conducted in future research.

## 5. Conclusions

The relationship between ants and actinobacteria is a fascinating area of research. Ants have been shown to host a diverse array of actinomycetes within their bodies, and these bacteria are believed to play crucial roles in ant health and behavior. Actinomycetes have been demonstrated to produce antibiotics that can help protect ants from pathogenic microbes. Based on this study, our findings revealed that the tropical ant species from Thailand carry a diverse range of actinomycete communities with antimicrobial properties. Here, we underscore the antimicrobial potential of actinomycetes isolated from ants and highlight the essential role of ants as a reservoir of bioactive compounds. The prevalence of *Streptomyces* strains among the isolated actinomycetes underscores their significance in this symbiotic relationship. Further investigations into the chemical diversity and mechanisms of action of these antimicrobial compounds hold promise for the development of novel therapeutics targeting Gram-positive pathogens and yeast infections.


