# Supplementary material for: Diversity and antimicrobial activity of the tropical ant-derived actinomycetes isolated from Thailand
Source: AIMS Microbiol. 2024 Jan 22;10(1):68–82. doi: 10.3934/microbiol.2024005 (PMC10955170; doi:10.3934/microbiol.2024005)
Supplement: Supplementary file 1 [file microbiol-10-01-005-s001.pdf]

Microbiology

AIMS Microbiology, 10(1): 68–82.
DOI: 10.3934/microbiol.2024005
Received: 02 November 2023
Revised: 30 December 2023
Accepted: 15 January 2024
Published: 22 January 2024

http://www.aimspress.com/journal/microbiology

Research article

Diversity and antimicrobial activity of the tropical ant-derived actinomycetes isolated from Thailand

Tuangrat Tunvongvinis[1], Weeyawat Jaitrong[2], Yudthana Samung[3], Somboon Tanasupawat[1,*], Wongsakorn Phongsopitanun[1,4,*]

[1] Department of Biochemistry and Microbiology, Faculty of Pharmaceutical Sciences. Chulalongkorn University, Bangkok 10330, Thailand
[2] Office of Natural Science Research, National Science Museum, 39, Moo 3, Khlong 5, Khlong Luang, Pathum Thani 12120, Thailand
[3] Department of Medical Entomology, Faculty of Tropical Medicine, Mahidol University, Bangkok 10400, Thailand
[4] Natural Products and Nanoparticles Research Units (NP2), Chulalongkorn University, Bangkok 10330, Thailand

* **Correspondence:** Email: wongsakorn.p@chula.ac.th; somboon.t@chula.ac.th.

Table S1. 16S rRNA gene sequence similarity and antimicrobial activity of the actinomycete isolates.

Ant species	Isolate number	Accession no.	Closet actinobacterial type strain	Similarity %	Antimicrobial activity (mm.)																	
					BS	SA	KR	EC	PA	SE	MRSA	KP	KA	*ST*	*S*	*SP*	*CA*	*CG*	*CGA*	*CT*	*CP*	*CPS*
Harpegnathos venator	HPV01	OQ559526	*Streptomyces rochei*	99.88%	-	-	-	-	-	-	-	-	-	-	-	-	-	-	-	-	-	-
	HPV02	OQ557939	*Amycolatopsis thermalba*	99.88%	-	-	-	-	-	-	-	-	-	-	-	-	-	-	-	-	-	-
	HPV04	OQ561248	*Nocardia cyriacigeorgica*	100.00%	-	-	-	-	-	-	-	-	-	-	-	-	-	-	-	-	-	-
	HPV06	OQ568319	*Streptomyces bikiniensis*	99.72%	-	-	17.00	-	-	-	-	-	-	-	-	-	-	-	-	-	-	-
Acanthomyrmex ferox	AC01	OQ559570	*Streptomyces exfoliates*	100.00%	-	-	-	-	-	-	-	-	-	-	-	-	-	-	-	-	-	-
	AC03	OQ557820	*Streptomyces rochei*	99.67%	-	-	-	-	-	-	-	-	-	-	-	-	-	-	-	-	-	-
	AC04	OQ557751	*Streptomyces rochei*	99.90%	-	-	-	-	-	-	-	-	-	-	-	-	-	-	-	-	-	-
Aphaenogaster feae	AF01	OQ565413	*Nocardia neocaledoniensis*	98.68%	-	-	-	-	-	-	-	-	-	-	-	-	-	-	-	-	-	-
	AF03	OQ558840	*Streptomyces rochei*	99.91%	-	-	-	-	-	-	-	-	-	-	-	-	-	-	-	-	-	-
Anochetus graeffei	AG02	OQ557750	*Streptomyces bikiniensis*	99.89%	-	-	-	-	-	-	-	-	-	-	-	-	-	-	-	-	-	-
	AG03	OQ561212	*Nocardia grenadensis*	98.25%	-	-	-	-	-	-	-	-	-	-	-	-	-	-	-	-	-	-
	AG04	OQ559525	*Streptomyces parvulus*	100.00%	-	-	-	-	-	-	-	-	-	-	-	-	-	-	-	-	-	-
	AG05	OQ561189	*Streptomyces parvulus*	99.47%	19.60	-	22.20	-	-	19.40	20.70	-	-	-	-	18.60	-	-	24.50	-	-	-
	AG06	OQ559661	*Streptomyces bikiniensis*	99.20%	-	-	-	-	-	-	-	-	-	-	-	-	-	-	-	-	-	-
	AG07	OQ559504	*Streptomyces atriruber*	99.77%	-	-	-	-	-	-	-	-	-	-	-	-	-	-	-	-	-	-

Continued on next page

Ant species	Isolate number	Accession no.	Closet actinobacterial type strain	Similarity %	Antimicrobial activity (mm.)																	
					BS	SA	KR	EC	PA	SE	MRSA	KP	KA	*ST*	*S*	*SP*	*CA*	*CG*	*CGA*	*CT*	*CP*	*CPS*
Camponotus lasiselene	CL01	OQ561249	*Streptomyces xylanilyticus*	99.35%	-	-	-	-	-	-	-	-	-	-	-	-	-	-	-	-	-	-
	CL02	OQ561251	*Streptomyces olivaceus*	99.79%	9.00	-	-	-	-	-	-	-	-	-	-	-	-	-	-	-	-	-
	CL05	OQ561252	*Streptomyces olivaceus*	99.07%	17.50	-	16.00	-	-	-	-	-	-	-	-	-	-	-	-	-	-	-
	CL10	OQ561253	*Streptomyces olivaceus*	99.93%	11.00	-	-	-	-	-	-	-	-	-	-	-	-	-	-	-	-	-
	CL11	OQ565676	*Streptomyces olivaceus*	99.24%	15.00	-	9.50	-	-	-	-	-	-	-	-	-	-	-	-	-	-	-
	CL12	OQ564377	*Streptomyces costaricanus*	100.00%	17.50	15.50	20	-	-	14.30	11.40	-	-	-	-	12.70	11.50	10.80	15.55	10.40	-	11.70
	CL13	OQ559423	*Streptomyces xylanilyticus*	99.74%	-	-	-	-	-	-	-	-	-	-	-	-	-	-	-	-	-	-
	CL14	OQ559568	*Streptomyces olivaceus*	99.89%	-	-	-	-	-	-	-	-	-	-	-	-	-	-	-	-	-	-
	CL15	OQ559566	*Streptomyces tendae*	100.00%	-	-	-	-	-	-	-	-	-	-	-	-	-	-	-	-	-	-
	CL17	OQ565633	*Streptomyces murinus*	100.00%	19.00	18.00	23.00	-	-	15.35	15.65	-	-	-	-	15.50	9.50	-	12.00	9.40	-	10.20
	CL18	OQ561295	*Streptomyces olivaceus*	99.52%	18.00	-	16.50	-	-	-	-	-	-	-	-	-	-	-	-	-	-	-
	CL19	OQ561296	*Streptomyces olivaceus*	99.86%	17.50	-	15.00	-	-	-	-	-	-	-	-	-	-	-	-	-	-	-
	CL20	OQ561298	*Streptomyces costaricanus*	100.00%	19.00	20	25.00	-	-	18.60	16.20	-	-	-	-	18.10	-	-	16.25	-	-	-
	CL23	OQ557997	*Streptomyces olivaceus*	99.93%	16.00	-	11.00	-	-	-	-	-	-	-	-	-	-	-	-	-	-	-
	CL25-3	ON520696	*Streptomyces aculeolatus*	99.84%	8.70	-	-	-	-	10.50	8.55	-	-	-	-	-	-	-	-	-	-	-
	CL27-1	OQ561299	*Streptomyces diacarni*	99.92%	-	-	-	-	-	-	-	-	-	-	-	-	-	-	-	-	-	-
	CL27-2	ON520694	*Streptomyces tendae*	100.00%	-	-	-	-	-	-	-	-	-	-	-	-	-	-	-	-	-	-
	CL30	OQ559392	*Streptomyces olivaceus*	99.89%	-	-	-	-	-	-	-	-	-	-	-	-	-	-	-	-	-	-
Centromyrmex feae	CF01	OQ560998	*Streptomyces zaomyceticus*	99.81%	-	-	-	-	-	-	-	-	-	-	-	-	-	-	-	-	-	-
Ectomomyrmex astutus	EA01	ON514566	*Nocardia asteroides*	99.92%	-	-	-	-	-	-	-	-	-	-	-	-	-	-	-	-	-	-

Continued on next page

Ant species	Isolate number	Accession no.	Closet actinobacterial type strain	Similarity %	Antimicrobial activity (mm.)																	
					BS	SA	KR	EC	PA	SE	MRSA	KP	KA	*ST*	*S*	*SP*	*CA*	*CG*	*CGA*	*CT*	*CP*	*CPS*
Echinopla striata	EC01	OQ557514	*Streptomyces violaceus*	100.00%	-	-	-	-	-	-	-	-	-	-	-	-	-	-	-	-	-	-
	EC03	OQ559327	*Streptomyces violaceus*	99.89%	-	-	-	-	-	-	-	-	-	-	-	-	-	-	-	-	-	-
Gnamptogenys coxalis	GC01	OQ558001	*Streptomyces araujoniae*	99.72%	-	-	-	-	-	-	-	-	-	-	-	-	-	-	-	-	-	-
	GC02	OQ559659	*Streptomyces rochei*	100.00%	-	-	-	-	-	-	-	-	-	-	-	-	-	-	-	-	-	-
Lioponera sp.	LI01	OQ557493	*Amycolatopsis australiensis*	99.65%	-	-	-	-	-	-	-	-	-	-	-	-	-	-	-	-	-	-
	LI03	OQ559622	*Streptomyces tendae*	99.27%	7.50	-	10.60	-	-	-	-	-	-	-	-	-	-	-	-	-	-	-
	LI04	OQ557483	*Streptomyces atriruber*	99.10%	-	-	-	-	-	-	-	-	-	-	-	-	-	-	-	-	-	-
Polyrchachis laevissima	LKA01	ON514610	*Streptomyces tendae*	100.00%	-	-	-	-	-	-	-	-	-	-	-	-	-	-	-	-	-	-
	LKA04	OQ557998	*Streptomyces parvulus*	100.00%	19.00	17.25	23.50	-	-	21.00	18.70	-	-	-	-	18.30	-	-	23.00	-	-	-
	LKA08	OQ560484	*Streptomyces parvulus*	100.00%	13.35	10	16.30	-	-	10.65	13.10	-	-	-	-	11.45	-	-	10	-	-	-
Odontomachus simillinus	ODS04-1	OQ557462	*Streptomyces monticola*	99.76%	-	-	-	-	-	-	-	-	-	-	-	-	-	-	-	-	-	-
	ODS05-4	ON514556	*Streptomyces lichenis*	98.85%	-	-	-	-	-	-	-	-	-	-	-	-	-	-	-	-	-	-
	ODS09	OQ559565	*Streptomyces griseourubiginosus*	99.78%	-	-	-	-	-	-	-	-	-	-	-	-	-	-	-	-	-	-
	ODS10	OQ557452	*Streptomyces lusitanus*	99.65%	-	-	-	-	-	-	-	-	-	-	-	-	-	-	-	-	-	-
	ODS11	ON514608	*Micromonospora citrea*	99.71%	-	-	-	-	-	-	-	-	-	-	-	-	-	-	-	-	-	-
	ODS15	ON514562	*Nocardia thailandica*	100.00%	-	-	-	-	-	-	-	-	-	-	-	-	-	-	-	-	-	-
	ODS15-2	OQ559560	*Streptomyces tendae*	100.00%	-	-	-	-	-	-	-	-	-	-	-	-	-	-	-	-	-	-
	ODS18	OQ557448	*Streptomyces sioyaensis*	100.00%	-	-	11.75	-	-	-	9.90	-	-	-	-	-	-	-	-	13.30	23.60	-
	ODS20	ON514585	*Streptomyces parvulus*	99.93%	14.80	-	16.15	-	-	10.50	14.70	-	-	-	-	14.00	-	-	18.10	-	-	-
	ODS25	ON514559	*Streptomyces lusitanus*	98.07%	11.70	-	-	-	-	8.20	12.75	-	-	-	-	8.45	-	-	-	-	-	-
	ODS28	OQ559042	*Streptomyces marinus*	97.12%	-	-	-	-	-	-	-	-	-	-	-	8.55	-	-	-	-	-	-

Continued on next page

Ant species	Isolate number	Accession no.	Closet actinobacterial type strain	Similarity %	Antimicrobial activity (mm.)																	
					BS	SA	KR	EC	PA	SE	MRSA	KP	KA	*ST*	*S*	*SP*	*CA*	*CG*	*CGA*	*CT*	*CP*	*CPS*
Parasyscia sp.	PA01	OQ559326	*Streptomyces drozdowiczii*	100.00%	-	-	-	-	-	-	-	-	-	-	-	-	-	-	-	-	-	-
	PA02	OQ559126	*Streptomyces bacillaris*	100.00%	-	-	-	-	-	-	-	-	-	-	-	-	-	-	-	-	-	-
	PA03	OQ559115	*Streptomyces microflavus*	100.00%	8.65	-	10.00	-	-	11.75	10.00	-	-	-	-	-	-	-	12.50	-	-	-
	PA05	OQ559106	*Streptomyces bacillaris*	100.00%	-	-	-	-	-	-	-	-	-	-	-	-	-	-	-	-	-	-
	PA06	OQ559107	*Streptomyces ambofaciens*	99.56%	-	-	-	-	-	-	-	-	-	-	-	-	-	-	-	-	-	-
Diacamma orbiculatum	RAB12	ON514568	*Streptomyces ardesiacus*	99.71%	14.40	13.70	-	-	-	16.75	14.55	-	-	-	-	-	-	-	-	-	-	-
	RAB13	ON514567	*Streptomyces albidoflavus*	99.54%	-	-	-	-	-	-	-	-	-	-	-	-	-	-	-	-	-	-
	RAB19	OQ561245	*Nocardia rhizosphaerihabitans*	99.04%	-	-	-	-	-	-	-	-	-	-	-	-	-	-	-	-	-	-
	RCB08	OQ558000	*Streptomyces djakartensis*	99.00%	-	-	-	-	-	-	-	-	-	-	-	-	-	-	-	-	-	-
	RCB10	ON519077	*Streptomyces drozdowiczii*	99.69%	-	-	-	-	-	-	-	-	-	-	-	-	-	-	-	-	-	-
	RCB24	OQ558003	*Streptomyces viridiviolaceus*	99.75%	-	-	-	-	-	-	-	-	-	-	-	-	-	-	-	-	-	-
Stigmatomma reclinatum	SR01	OQ559621	*Streptomyces ardesiacus*	99.51%	-	-	-	-	-	-	-	-	-	-	-	-	-	-	-	-	-	-
	STR09	OQ559556	*Streptomyces atriruber*	99.56%	-	-	-	-	-	-	-	-	-	-	-	-	-	-	-	-	-	-
Syscia chaladthanyakiji	SY02	OQ559571	*Nocardia thailandica*	98.90%	-	-	-	-	-	-	-	-	-	-	-	-	-	-	-	-	-	-
Tetraponera nigra	TEN01	OQ561246	*Streptomyces costaricanus*	100.00%	9.00	-	-	-	-	-	12.20	-	-	-	-	-	-	-	-	-	-	-
Tetraponera rufonigra	TER04	ON515460	*Nocardia aurantia*	99.58%	-	-	-	-	-	-	-	-	-	-	-	-	-	-	-	-	-	-

Abbreviations: BA, *Bacillus subtilis*; SA, *Staphylococcus aureus*; KR, *Kocuria rhizophila*; EC, *Escherichia coli*; PA, *Pseudomonas aeruginosa*, SE, *Staphylococcus epidermidis*; MRSA, Methicillin-resistant *Staphylococcus aureus*; KP, *Klebsiella pneumoniae*; KA, *Klebsiella aerogenes*; ST, *Salmonella typhi*; S, *Shigella* sp.; SP, *Streptococcus pyogenes*; CA; *Candida albicans*; CG, *Candida glabrata*; CGA, *Candida guilliermondii*, CT, *Candida tropicalis*; *Candida parapsilosis*; CPS, *Candida pseudotropicalis*

AIMS Press

© 2024 the Author(s), licensee AIMS Press. This is an open access article distributed under the terms of the Creative Commons Attribution License (http://creativecommons.org/licenses/by/4.0)
